# Persistence of human Aichi virus infectivity from raw surface water to drinking water

**DOI:** 10.1128/aem.01189-24

**Published:** 2024-12-31

**Authors:** Khira Sdiri-Loulizi, Amira Khachou, Stephanie Lemaire, Jean-Baptiste Bour, Siwar Ayouni, Jérôme Kaplon, Nabil Sakly, Mahjoub Aouni, Gael Belliot, Alexis de Rougemont

**Affiliations:** 1 Centre National de Référence des virus des gastro-entérites, Centre Hospitalier Universitaire Dijon Bourgogne 36659 , Dijon, France; 2 Laboratoire des maladies transmissibles et substances biologiquement actives, Faculté de Pharmacie, Université de Monastir 37966 , Monastir, Tunisia; 3 Institut Supérieur de Biotechnologie de Béja, Université de Jendouba 201303 , Jendouba, Tunisia; 4 UMR PAM A 02.102 Procédés Alimentaires et Microbiologiques, Université Bourgogne Europe / INRAe / L’Institut Agro Dijon 27011 , Dijon, France; 5 Laboratoire de Virologie-Sérologie, Centre Hospitalier Universitaire Dijon Bourgogne 36659 , Dijon, France; 6 Laboratoire d’Immunologie, Centre Hospitalier Universitaire Fattouma-Bourguiba, Monastir, Tunisia; Colorado School of Mines, Golden, Colorado, USA

**Keywords:** AiV-1, Aichi virus, ICC-RT-qPCR, infectious viral particles, virus quantification, wastewater, drinking water, surface water

## Abstract

Human Aichi virus 1 (AiV-1) is a water- and food-borne infection-associated picornavirus that causes gastroenteritis in humans. Recent studies on environmental waters showed a high frequency and abundance of AiV-1, suggesting that it might be an appropriate indicator of fecal contamination. We screened 450 surface and drinking water samples from a Tunisian drinking water treatment plant (DWTP) and the Sidi Salem dam for AiV-1 by real time reverse transcriptase PCR (RT-qPCR). The persistence of infectious particles was evaluated using an integrated cell culture approach coupled with quantitative molecular detection (ICC-RT-qPCR). In all, 85 (18.9%) water samples were positive for AiV-1 with viral loads ranging from 0.47 to 11.62 log_10_ cp/L and a median of 4.97 log_10_ cp/L, including 30/100 raw, 18/50 decanted, 14/50 flocculated, 9/100 treated, 1/50 tap, and 13/100 surface water samples. Of these, 15 (17.6%) samples contained infectious AiV-1 genotype A particles, including five raw, four decanted, one surface, three flocculated, and two treated water samples. Our data suggest that the persistence of infectious AiV-1 particles in environmental waters might represent a potential threat to public health. This study also indicates that the ICC-RT-qPCR is a practical tool for monitoring human waterborne viral risk in aquatic environments.

**IMPORTANCE:**

Human Aichi virus 1 (AiV-1) is a water- and food-borne infection-associated picornavirus that causes gastroenteritis in humans. Its high frequency and abundance in environmental waters would suggest that it might be an appropriate indicator of fecal contamination. The analysis of surface and drinking water samples from a Tunisian drinking water treatment plant (DWTP) and the Sidi Salem dam using an integrated cell culture approach coupled with a quantitative molecular detection (ICC-RT-qPCR) confirmed the persistence of infectious AiV-1 particles in samples at all stages of the treatment process, except in tap water. This suggests that the persistence of AiV-1 infectivity in environmental waters might represent a potential threat to public health. This study also indicates that the ICC-RT-qPCR is a practical tool for monitoring human waterborne viral risk in aquatic environments.

## INTRODUCTION

Waterborne viral infection is one of the most important causes of human morbidity, and related diseases continue to have public health and socioeconomic implications worldwide. Diarrheal diseases are reported as the leading cause of mortality among children under 5 years old, representing 63% of the global burden (1). According to 2018 WHO reports, diarrheal diseases are responsible for the deaths of approximately 525,000 children under 5 years old every year, 90% of which occur in developing countries (2, 3) where poor sanitation and insufficient potable water supply are key factors (4, 5). Inadequate water and sanitation affect nearly 750 million people around the world (6) and have long been associated with diarrhea (7, 8) in low-income countries, especially on the African continent, where climate change is argued to have the greatest impact on water, food security, and public health.

**Fig 1:**
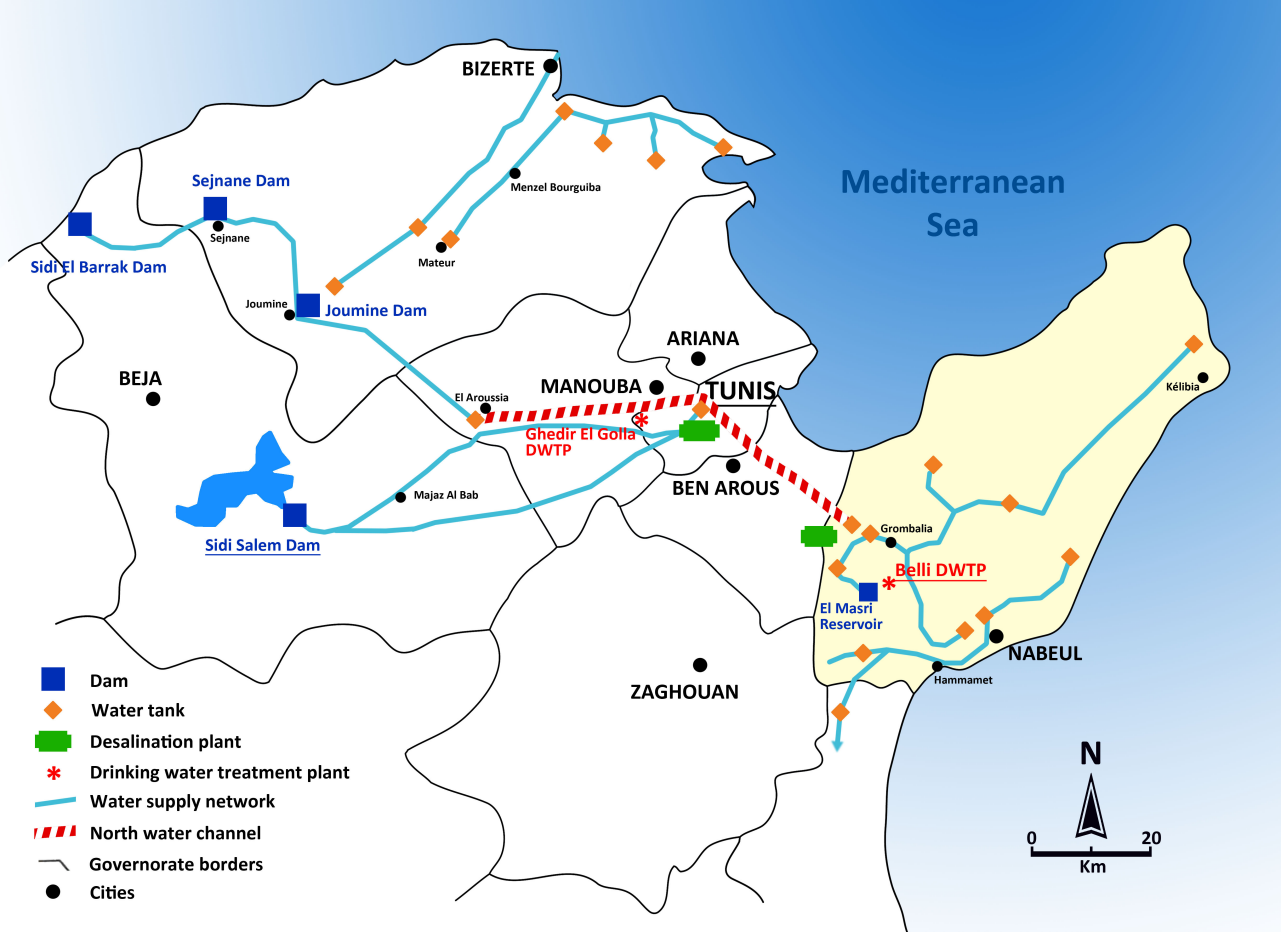
Map of the drinking water network in northern Tunisia during the study period (adapted from Chabbi et al. [10]).

In Tunisia, which is one of the most drought-stressed Mediterranean countries, the per capita renewable water availability is 486 m^3^, well below the average of 1,200 m^3^/capita for the Middle East and North Africa region. According to the Joint Monitoring Program by WHO and UNICEF, Tunisia has achieved the highest access rates to water supply and sanitation services in the Middle East and North Africa. The national water service SONEDE (Société Nationale d'Exploitation et de Distribution des Eaux) operates throughout Tunisia. It is the only company to manage the national water supply and provides drinking water to 100% of the urban population and around 50% of the rural population. The sources of drinking water differ from one area to another according to the geographical and climatic characteristics, and the development of their water requirements (Fig. 1). The Tunisian strategy in terms of water resources management, particularly in drinking water, is based on a system of water transfer from the areas with the most abundant resources (North and North*-*West) toward the areas with the most significant consumption (Center and South). The SECADENORD water company (Société d'Exploitation du Canal et des Adductions des Eaux du Nord) ensures the management and maintenance of the North-West part of the network of water transfer (pipes and channels) between different Tunisian areas. The role of this company is to mobilize and transfer northern Tunisian waters from the extreme North-West to the users located in the North-East, Centre, and South of the country (areas of Greater Tunis, Coastal Sahel, Kairouan, Sfax, and Sidi Bouzid) where there is a shortage of potable water. Most of the water in the northern region comes from the Sidi Salem, Joumine, Sejnane, and Sidi El Barrak dams, which have an interconnected system (9) (Fig. 1).

SONEDE and the Tunisian Ministry of Agriculture, Water Resources, and Fisheries are responsible for monitoring the drinking water quality and for assessing the bacteriological and physicochemical qualities of the water from production to distribution (11). The Tunisian standards for microbiological water quality only include bacterial fecal indicators such as fecal coliforms and streptococci (12, 13). However, these indicators are not sufficient because they do not accurately assess the presence of viruses and hardly determine the roots of the fecal contamination (14, 15). Several studies have shown viral water contamination, particularly in wastewater, making Tunisia an endemic area for the hepatitis A virus (16, 17), enterovirus (18, 19), and gastroenteritis viruses such as group A rotavirus, norovirus, astrovirus, enteric adenovirus F40/F41, and Aichi virus AiV-1 (20–25). While epidemiological and clinical data have been published regarding gastroenteritis viruses and the possible link between water contamination and viral infantile diarrhea (24), the role of water as a virus disseminator or an epidemic booster in winter enteric virus outbreaks has yet to be demonstrated, especially in countries with efficient water treatment facilities. This is particularly due to the distance between the place of contamination and the place where the disease is reported, the variability of the virus incubation period, and the frequent under-reporting of cases within the epidemiological surveillance systems. Nevertheless, to date, no studies have investigated enteric viruses in surface and drinking water resources in Tunisia.

Human Aichi viruses (AiV-1, also written AiV-A1) were first described in 1991 (26) and subsequently classified in 1999 by the ICTV as species *Aichivirus A* of the new genus *Kobuvirus* from the family *Picornaviridae*. Like many other picornaviruses, AiV-1 is a small round non-enveloped virus with an icosahedral capsid of 30 nm diameter with receptor binding sites and harboring a single positive strand RNA genome of 7.3 kb (27). Aichi virus resists very low pH conditions and conventional methods of inactivation, including alcohol, chlorine, heat, or non-ionic detergents (28). To date, three genotypes have been described among AiV-1 strains: genotypes A, B, and C (29). Genotype A is the most widespread in the world where its seroprevalence varies between 25% and 60% in children under 10 and between 80% and 99% in adult populations, which is indicative of a large exposure (30–35). Several environmental studies showed that AiV-1 is a viral pathogen associated with environmental contamination and water- and food-borne infections (25, 36–42). In our previous studies, we showed that AiV-1 was the second most frequently detected enteric virus in Tunisian sewage, a possible source of contamination of irrigation or drinking water, and was also associated with severe acute diarrhea in Tunisian children (25). Although real-time PCR tests can detect the AiV-1 genome, they are unable to discriminate between viable and inactivated virions. Therefore, the presence of a large quantity of viral particles does not necessarily correlate with a potential threat to human health.

In this study, we investigated the presence of human Aichi viruses in surface and drinking water samples collected during a 2-year period from October 2011 to November 2013 in Tunisia and characterized the circulating strains using molecular biology techniques. We then determined the infectivity of these AiV-1 strains using an integrated cell culture method coupled with a quantitative real-time PCR (ICC-RT-qPCR). To our knowledge, this is the first study in the literature to report the detection of infectious AiV-1 in surface and drinking water.

## MATERIALS AND METHODS

### Water treatment plant settings and collections

Located in the northeast of the country and surrounded by the Mediterranean on three sides with a coastline of 300 km, the governorate of Cap-Bon has 787,920 inhabitants (2014 census). Its climate is semi-arid with a mean annual precipitation of 400 mm and a mean temperature of 19°C (43). The regional water supply is derived from four principal sources: surface water from local dams and groundwater, which are utilized for irrigation purposes, treated wastewater, and the transfer of surface water from the Medjerda River in northern Tunisia (Fig. 1). The 160 km long Medjerda*-*Cap*-*Bon canal feeds large irrigated perimeters in the Cap*-*Bon area*,* and supplies drinking water to major coastal cities.

Water was sampled at the water pumping station of the Belli drinking water treatment plant (DWTP) near Nabeul that treats surface water coming from dams of high rainfall areas of northern Tunisia via the canal and collected at the El Masri damned reservoir. The Belli DWTP station uses physio-chemical treatments including successive steps of aeration, coagulation-flocculation (chemical coagulants such as aluminum sulfate and polyelectrolytes to remove color, turbidity, algae, and other microorganisms from surface waters, and to cause the formation of precipitates or flocs), decantation (collection of the suspended solids, aggregation of the flocs, concentration, and elimination of sludge), filtration (algae and microorganism are removed from raw waters by slow sand filters, while pumps and air boosters ensure backwashing), pre-chlorination (chlorine injection to reduce the content of organic matters, and to eliminate the proliferation of algae), and final physicochemical disinfection (injection of optimal quantities of bleach to destroy persistent micro-organisms and to reduce possible network contaminations).

**Fig 2:**
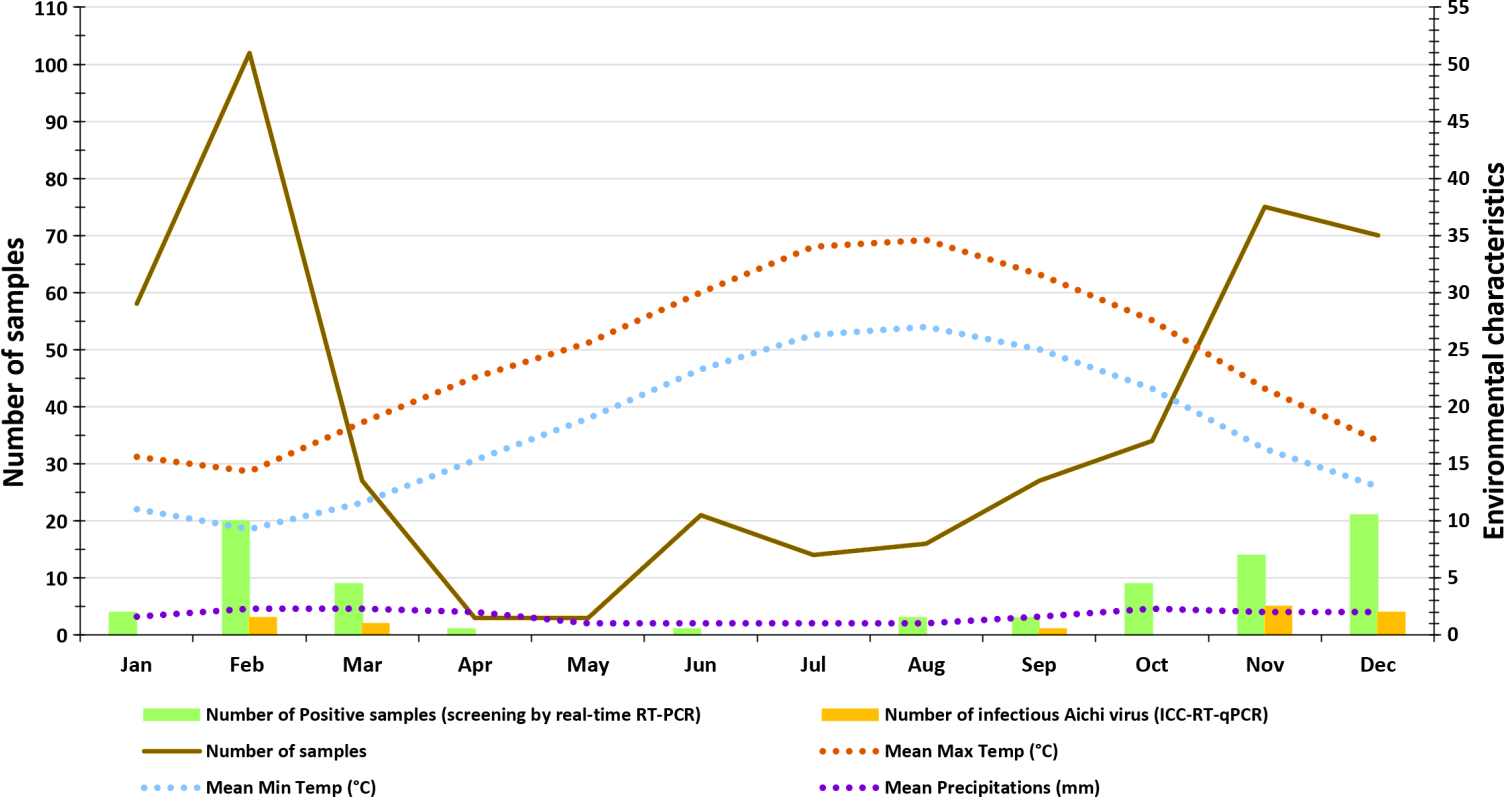
Average monthly number of cases of AiV-1 contamination of water and average environmental characteristics in Tunisia.

From October 2011 to November 2013, samples of 2 L of water were collected every month using sterile plastic flasks and stored up to 48 h at 4°C until further processing (Fig. 2). In all, 450 samples were collected during the period study. At the Belli DWTP, 300 water samples were collected from four main points of the treatment chain: 100 samples of raw water (RW) at the pumping station entry, 50 of decanted water (DW), 50 of flocculated water (FW), and 100 of treated water (TW) at the pumping station outlet. In addition, 100 samples of surface water (SSD-W) were collected from Sidi Salem dam and 50 of treated tap water (TTW) from the water distribution network in Nabeul. All samples were analyzed for AiV-1 as described below.

### Water sample treatment

Within 24 h after collection, each 2 L water sample was processed for virus concentration using an adsorption-elution method (44, 45) followed by a polyethylene glycol precipitation (25, 46), which has an estimated recovery rate of 69.5% ± 8.5%. Briefly, the samples were acidified at pH 3.5 with 12 N HCl and treated with sterile MgCl_2_ at 25 mM for 45 min with periodic stirring to coagulate particles and microorganisms, and positively charge virus particles. Virions were then adsorbed onto a sterile electronegative cellulose nitrate membrane filter (Millipore HA; 0.45 mm pore size; 47 mm diameter) at a flow rate of 100 mL/min. Viral fraction was then eluted with 10 mL of 3% beef extract solution at pH 9 (ref. LP029; Oxoid Ltd., England). The eluates were clarified by centrifugation at 6,000 × *g* for 45 min at 4°C prior to precipitation using polyethylene glycol 6000 (PEG 6000). After an overnight incubation at 4°C, the samples were centrifuged at 10,000 × *g* for 45 min at 4°C, and the resulting pellet was resuspended in 2 mL of phosphate-buffered saline at pH 7. The 1,000-fold concentrated samples were then stored at −80°C until RNA extraction.

### Sample screening and virus quantification

Viral RNA was extracted from a volume of 1 mL of concentrated water samples using a NucliSens EasyMAG automated extractor (bioMérieux, France), according to the manufacturer’s instructions. Sample RNAs were eluted in a final volume of 110 µL. AiV-1 screening and quantification RT-qPCRs were performed using a 5′UTR targeting primer set, as previously described (47). An AiV-positive control plasmid (pGEM-AiV) was kindly provided by Dr. Sébastien Wurtzer from Eau de Paris (Paris, France), as previously described (40), to produce RNA transcripts using the RiboMAX T7 Large Scale RNA Production System kit (Promega, USA) according to the manufacturer’s recommendations. The pGEM-AiV-1 was linearized using the ScaI restriction enzyme (Sigma-Aldrich, USA), purified, and quantified with a Nanodrop ND-1000 spectrophotometer (LabTech, France) prior to transcription. Serial RNA transcript concentrations, ranging from 1.10^1^ to 1.10^10^ genome copies per µL (gc/µL), were used to determine the end-point limit of detection (LoD) and the linearity of the assay (Fig. S1). The standard quantification curve showed a linear correlation with a LoD of 2.2 × 10^4^ copies per liter (cp/L), which enabled us to detect the presence of low viral RNA quantities in environmental samples, including surface water and drinking water.

### Integrated cell culture and quantitative reverse transcription PCR assay

An integrated cell culture reverse transcriptase quantitative PCR (ICC-RT-qPCR) was set to quantify infectious AiV-1 virions present in water samples. The cell culture system was first assessed to determine its reproducibility and repeatability using the AiV-1 A846/88 strain, which was kindly provided by Dr. Teruo Yamashita (Aichi Prefectural Institute of Public Health, Japan). Vero cells were seeded in 24-well tissue culture plates at a density of 3 × 10^6^ cells per well in MEM (Gibco, Thermo Fischer Scientific, USA) supplemented with 10% fetal calf serum (Eurobio Scientific, France), 1% Eagle’s nonessential amino acids (100×; Eurobio Scientific, France), 1% glutamine (200 mM; Sigma-Aldrich, USA), and 1% antibiotics (penicillin, 5,000 UI/mL; streptomycin, 5,000 µg/mL), as described previously (48–50). Plates were incubated for 24 h at 37°C in 5% CO_2_ prior to inoculation. AiV-1 was grown for 48 h then recovered from whole culture lysates, divided into aliquots at 10^8.5^ TCID_50_/mL, and stored at −80°C until further use, as previously described (30). Serial dilutions of 10^−1^ to 10^−4^ of 1 mL AiV-1 stock were then used as positive controls in PCRs.

Inoculation procedures of water samples for ICC-RT-qPCR were conducted as previously described (51, 52). Vero cells were grown for 24 h, in the same condition as mentioned above, prior to inoculation of 200 µL of each water sample in triplicate. Both cell lysates and supernatants were harvested at t0 and t48 hours post-infection (hpi) and immediately stored at −20°C for further analysis. RNA extraction was then performed on 1,000 µL of lysate before quantification of infectious particles, as described above.

### Sequencing and virus characterization

AiV-1-positive samples were amplified using the Qiagen One Step RT-PCR kit (Qiagen, Germany), which generated a 519 bp fragment located at the 3CD junction as previously described (53). The amplicons were then sequenced on an ABI 3130XL sequencer (Life Technology, Thermo Fischer Scientific, USA) using the ABI Prism Big Dye Terminator cycle v3.1 sequencing kit (Life Technology, Thermo Fischer Scientific, USA). Generated sequences were aligned on MEGA X software using the ClustalW tool (54) and then compared with GenBank reference sequences using BLAST. A phylogenetic tree based on 3CD region sequences was constructed using the maximum-likelihood method with the Kimura 2-parameter substitution model and a discrete gamma distribution and invariable sites. Bootstrap values were calculated for 1,000 replicates and are indicated at each node.

### Statistical analysis

Kruskal-Wallis one-way analysis of variance tests and multicomponent analysis were conducted using SPSS v.19 software (IBM, USA). Wilcoxon signed rank tests were also performed to compare AiV-1 viral loads at t0 and t48 hpi. All *P* values ≤ 0.05 were considered statistically significant.

## RESULTS

### Water sample AiV-1 screening

The results of the AiV screening are presented in Table 1. The AiV-1 genome was detected in 85/450 (18.9%) water samples, with a median viral load of 4.97 log_10_ cp/L (Ct range = [4.01;39.43]), of these, 30/100 RW, 18/50 DW, 14/50 from FW, 9/100 TW, 1/50 TTW, and 13/100 SSD-W (Table S1). There was no significant difference in viral loads between different water origins (*P* = 0.522).

**TABLE 1:** AiV-1 screening and ICC-RT-qPCR assay results by water sample origin^*a*^

	Origins of water samples^*b*^					
	RW	DW	FW	TW	TTW	SSD-W
Number of samples (*n* = 450)	100	50	50	100	50	100
RT-qPCR screening						
AiV-1 positive samples (*n* = 85)	30 *35.3%*	18 *21.2%*	14 *16.4%*	9 *10.6%*	1 *1.2%*	13 *15.3%*
Median viral loads [range] (log_10_ cp/L)	6.98	3.27	8.28	4.15	7.68	4.26
	[0.47;10.83]	[1.32;11.62]	[1.03;10.72]	[1.16;8.60]	–^*c*^	[1.68;9.92]
ICC-RT-qPCR assays						
Number of infectious AiV-1 samples (*n* = 15)	5 *33.3%*	4 *26.6%*	3 *20.0%*	2 *13.3%*	0 –	1 *6.6%*
Mean viral loads [range] (log_10_ cp/L)						
t0 hpi	6.18	6.71	3.13	6.02	–	3.56
	[nd;6.88]	[nd;7.06]	[nd;2.70]	[nd;6.32]	–	–
t48 hpi	9.43	9.24	4.50	9.49	–	4.00
	[4.30;10.08]	[4.40;9.73]	[4.13;4.66]	[7.37;9.79]	–	–
Number of virus sequences (*n* = 12)	5 *41.6%*	2 *16.6%*	3 *25%*	1 *8.3%*	0	1 *8.3%*
GenBank accessing numbers	*#MG977515* *#MG977516* *#MG977518* *#MG977519* *#MG977522*	*#MG977520* *#MG977523*	*#MG977514* *#MG977521* *#MG977524*	*#MG977513*	–	*#MG977517*

^*a*^ Ct, cycle threshold; SD, standard deviation; cp/mL, copies per mL; nd, not detected.

^*b*^ RW, raw water; DW, decanted water; FW, flocculated water; TW, treated water; TTW, treated tap water; SSD-W, surface water from Sidi Salem dam.

^*c*^ "–" means either no data or no calculation.

In all, AiV-1 was significantly more frequently detected during the cold and rainy months (October to February), with 69 (81.2%) samples (median viral load = 5.38 log_10_ cp/L; range = [0.47;11.62]), than during the hot and dry months (May to September), with 7 (8.2%) samples (median viral load = 7.74 log_10_ cp/L; range = [1.03;10.83]) (*P* < 0.008), but no difference in viral loads depending on sampling month, temperature, and precipitation for each water origin (*P* = 0.20) (Fig. 2). Although AiV-1 detection was more frequent during rainy periods, with 57 (67.1%) positive samples, there was no significant statistical correlation between sample positivity and mean temperature (*rs* = −0.01) or mean precipitation (*rs* = 0.02). In addition, there was no significant difference when comparing viral loads depending on water origin (*P* = 0.522).

Average environmental characteristics of northern Tunisia such as rainfall (in mm), and mean maximal and minimal temperatures (in **°**C) were obtained from the National Institute of Tunisian Meteorology for the study period from 2010 to 2013. Of note, many regions in Tunisia suffered from massive unusual floods after torrential rainfall in October 2011, November 2011, February 2012, and September 2013. A record rainfall was observed in the North-West of Tunisia in October 2011: the day rainfall record on Sunday 30 October 2011 was 105 mm in Tabarka, 53 mm in Beja, and 70 mm in Tunis. The day rainfall record for November 2011 was 31 mm. Concerning the record rainfall in 2012, it rained 250 mm over 2 days from 22 to 23 February in the North (Bizerte) and in the North-West of Tunisia (Jendouba and Beja).

### AiV-1 infectivity persistence

**Fig 3:**
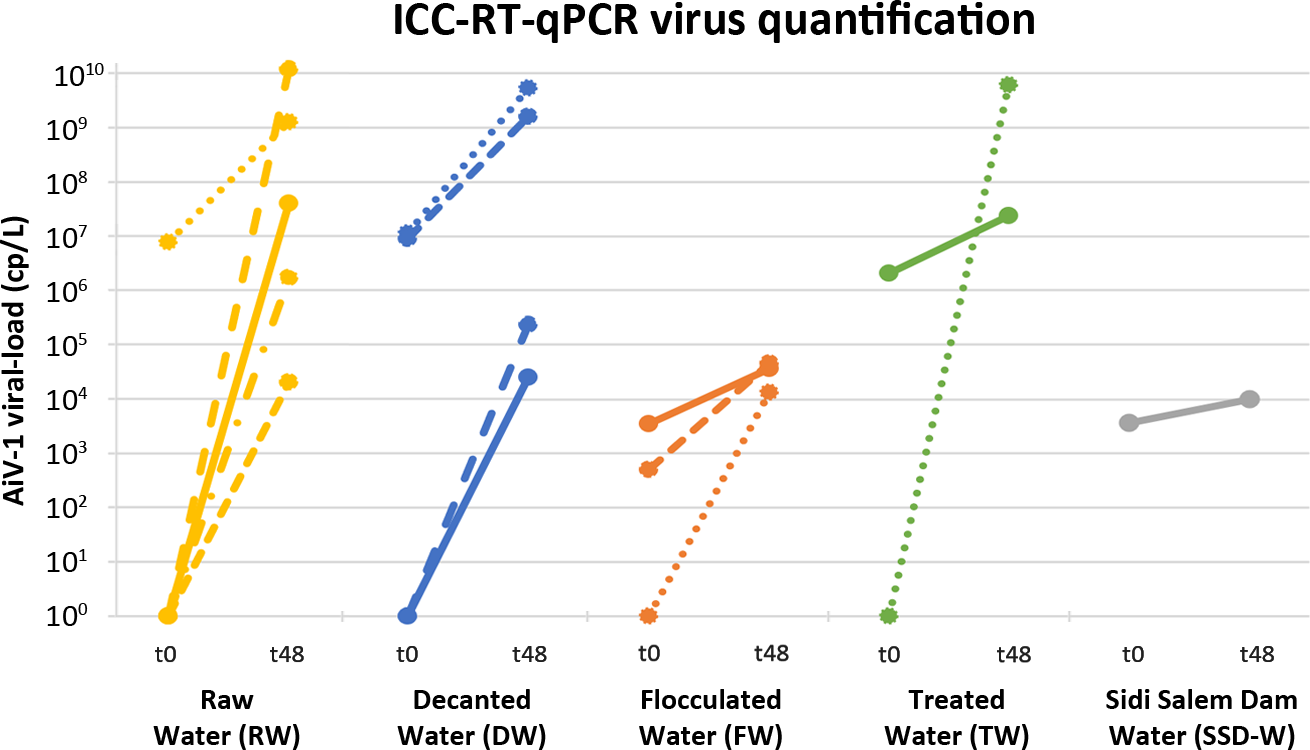
ICC-RT-qPCR virus quantification by water source.

The ICC-RT-qPCR results are presented in Table 1, and measured viral loads are shown for each water source in Fig. 3. All AiV-1*-*positive samples were tested to determine persistent virus infectivity by ICC-RT-qPCR. Of the 85 positive samples, 15 (17.6%) contained AiV-1 virions that were still infectious. The 15 water samples were collected during cold and dry months from September to March: one in September, five in November, four in December, three in February, and two in March (Fig. 2). The infectious samples originated from RW (*n* = 5), DW (*n* = 4), FW (*n* = 3), TW (*n* = 2) and one from SSD-W, but none from TTW, although one water sample initially tested positive. Interestingly, cytopathic effects in Vero cell culture were observed in 7 (46.7%) of the 15 samples after 72 hpi. AiV-1 strains are available upon request.

Depending on the water origin, the maximum measured viral loads ranged from 1.70 to 7.78 log_10_ cp/L. The highest viral loads at 48 hpi were detected in samples collected during November and December in RW (7.87 log_10_/L), DW (7.43 log_10_ cp/L), FW (2.36 log_10_ cp/L), and TW (7.49 log_10_ cp/L). No significant difference between water sample origins was found at t0 and t48 hpi in viral loads (*P* = 0.69 and *P* = 0.23, respectively).

### Sequencing and molecular typing

Of the 15 samples presenting infectious AiV-1 particles, only 12 could be characterized by genome sequencing depending on viral loads: RW (*n* = 5), DW (*n* = 2), FW (*n* = 3), TW (*n* = 1), and SSD-W (*n* = 1) (GenBank access numbers #MG977513 to #MG977524). A phylogenetic tree of partial AiV-1 3CD nucleotide sequences from Tunisian water samples compared with reference sequences from GenBank is shown in Fig. S2. All 12 virus strains belonged to genotype A according to the human Aichi virus classification (29, 35, 53). The sequence analysis and phylogenetic study of AiV-1 strains showed that our AiV-1 sequences were 100% identical and clustered with the Japanese A1258/1987 strain AB034649, with 100% nucleotide sequence similarity.

## DISCUSSION

So far, few studies around the world have undertaken a quantitative detection of human Aichi virus in different types of environmental water, particularly effluents, and none have investigated the persistence of infectivity. Here, we report the first evidence of the presence of infectious AiV-1 virions in water using the ICC-RT-qPCR method. The advantage of ICC-RT-qPCR is the ability to differentiate infectious from inactivated virions. To our knowledge, this is the first study assessing human Aichi virus infectivity in environmental samples.

Since its discovery, the human Aichi virus has been found to play a role in some acute gastroenteritis outbreaks in several regions of the world and has extensively been detected in various environmental samples, suggesting its potential as an environmental viral contamination marker (36). As AiV-1 has been linked to pediatric cases of severe acute gastroenteritis due to contamination of drinking water by sewage (55, 56), it is essential to monitor AiV-1 in the environment in order to better understand the epidemiology and to identify the viral contamination sources, provided it originated from the water network. Additionally, monitoring the water network might contribute to a more comprehensive picture of enteric viruses, including AiV-1, circulating within the population, as recently described for severe acute respiratory syndrome coronavirus 2 and other respiratory or enteric viruses (57–59).

The aim of the present study was to investigate the role of AiV-1 in waterborne epidemics in Tunisia through the persistence of its infectivity at all stages of water treatment, from raw water to tap water. AiV-1 genomes were detected in 85 (18.9%) of the 450 water samples that were tested (Table 1), most originating from four main water treatment stages at the Belli DWTP, from raw water (35.3%) to treated water (10.6%). AiV-1 was also detected for the first time in Tunisia in surface waters (15.2%), as reported in other places in the world (40, 60–62). It is worth mentioning that AiV-1 was also found in one (1.2%) sample of drinking water.

The present results are in accordance with previous studies which reported AiV-1 contamination ranging from 11% to 100% of surface and drinking waters, in ascending order, in France, Netherlands, the United States, Japan, Venezuela, Iran, and Nepal (37, 39–41, 60, 62–65). Detecting AiV-1 in all water samples, regardless of their origin, supports the proposition that AiV-1 could potentially serve as a viral indicator of water pollution. Indeed, previous environmental studies have reported higher detection rates for AiV-1 compared to other enteric viruses in various water types, including wastewater, groundwater, and river water (35, 37, 66). Furthermore, the presence of AiV-1 in Tunisian waters is consistent with the high AiV-1 seroprevalence in the Tunisian population (25) and its frequent detection in stool, sewage, and shellfish samples, as reported previously (25, 67, 68).

High AiV-1 viral loads were obtained from RW, from the pumping station inlet, DW, and TW samples collected at the DWTP. These viral concentrations were higher than those found in water samples from other countries studies. In Japan, although AiV-1 was found in 100% of river water samples, virus concentrations ranged from 2.94 to 4.30 log_10_ cp/L, greater than for other enteric viruses (62), and up to 4.0 log_10_ cp/L in surface water (63). In Nepal, Iran, and France, AiV-1 concentrations in river waters were usually estimated up to 8.92, 6.77, and 2.0 log_10_ cp/L, respectively (40, 64, 65). However, a study conducted in Nepal showed an AiV-1 concentration of 9.0 log_10_ cp/L in drinking tap water (65). These high AiV-1 concentrations could be the consequence of high levels of shedding by infected individuals (69) combined with a possible discharge of untreated wastewater directly into environmental water.

As expected, AiV-1 detection frequency was significantly higher in cold or rainy seasons than during dry or hot seasons (Fig. 2), which is consistent with the observed seasonality in the epidemiology of pediatric gastroenteritis in Tunisia (68). Studies conducted in Iran, Japan, and France have shown that the frequency and concentration of AiV-1 in river water are higher during periods of rainfall (62, 64, 70, 71). Indeed, the effects of precipitation on the occurrence of viruses in river or dam water, as a resource for drinking water, when impacted by sewer overflows could influence the tap water quality (40, 62). Overall, these findings may thus reflect a close relationship between the health status of the pediatric population in particular, and the level of viral contamination of surface and drinking water, since Aichi virus infections are mainly contracted during childhood, when around 70% of Tunisian children under 10 acquire specific antibodies against this virus (33). The monitoring of both wastewater and environmental water could provide valuable information on the circulation of human viruses within the population, and contribute to safeguarding public health.

Sequence and phylogenetic analysis of the 3CD nucleotide sequences of 12 strains showed that all strains belonged to genotype A, as observed in previous studies on surface water (37, 39). Indeed, genotype A has been identified more frequently in water and environmental samples than genotype B (35), while genotype C has never been isolated from wastewater. While genotype A has been detected in sewage, clinical samples, and shellfish in Tunisia since 2003 (25, 68), genotype B has only recently been detected in clinical and wastewater samples, consistent with other parts of Africa such as Ethiopia, South Africa, Burkina Faso, and Senegal (67, 72–75).

Nucleotide sequences of our AiV-1 strains had an identity similar to those circulating in the Tunisian pediatric population in the same period (68), with a high degree of sequence similarity in the 3C-3D junction gene (94.6% and 100% identity). Moreover, these AiV-1 strains shared 95% to 96.1% nucleotide sequence identity with sewage strains, and 94.2% to 95.4% with shellfish strains found in Tunisia (25, 68). This is further supported by the fact that AiV strains from this study have 100% homology with previously described isolates from Japan, Bangladesh, Vietnam, France, or Sweden (29, 76, 77). This suggests that the 3CD gene sequence is well conserved worldwide. Additionally, the high degree of similarity between AiV-1 strains detected in surface water and those in human infections and sewage strongly suggests the epidemiological link between soiled water and AiV-1-infected individuals.

As suggested above, human Aichi viruses could be an interesting indicator of human-specific fecal viral pollution in the environment. A better understanding of the persistence of infectious viruses over the course of water and wastewater treatment processes would enable public authorities to better manage the health risks associated with recycling treated wastewater and discharging it into the environment. However, to assess the risk of viral water contamination in developing countries, further studies are required to assess the prevalence and infectivity of enteric viruses in water. Therefore, it is essential to monitor water quality to understand seasonal variations in water quality, waterborne disease transmission, and the future implications of climate change and public health. This study confirmed the presence of AiV-1 in environmental, surface, and drinking water, with infectious particles observed in treated water from DWTP. These findings highlight the potential public health risks posed by the release of these viruses into the environment. Further investigations will be needed to better evaluate the real impact of AiV-1 in environmental water and to fully understand AiV-1-related diarrheal disease and epidemiology, especially in children. This data will be required to determine the need for specific preventive measures.

## SUPPLEMENTAL MATERIAL

Supplemental material aem.01189-24-s0001.pdf Figures S1 and S2; Table S1.

ASM does not own the copyrights to Supplemental Material that may be linked to, or accessed through, an article. The authors have granted ASM a non-exclusive, world-wide license to publish the Supplemental Material files. Please contact the corresponding author directly for reuse.
