# Supplementary material for: Persistence of human Aichi virus infectivity from raw surface water to drinking water
Source: Appl Environ Microbiol. 2024 Dec 31;91(1):e01189-24. doi: 10.1128/aem.01189-24 (PMC11784453; doi:10.1128/aem.01189-24)
Supplement: Supplemental material — Figures S1 and S2; Table S1. [file aem.01189-24-s0001.pdf]

SUPPLEMENTAL DATA

Figure S1. AiV-1 quantification standard curve

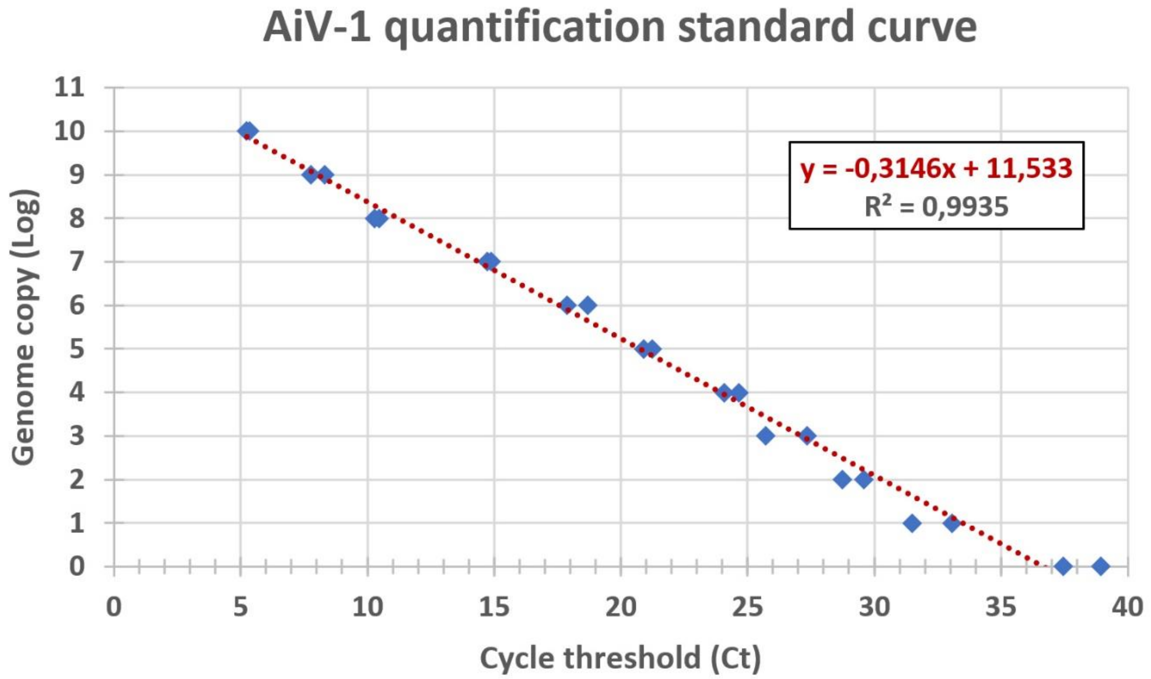

The determination of the standard curve for AiV-1 quantification by RT-qPCR was performed in duplicate using serial dilutions of viral RNA ranging from 1 to 1.10^{10} genome copies per reaction.

cp/µL (n)	gc/µL (Log_{10})	Mean Ct
1	0	38.2 ±1.1
10	1	32.3 ±1.1
100	2	29.2 ±0.6
1,000	3	26.5 ±1.2
10,000	4	24.4 ±0.4
100,000	5	21.1 ±0.2
1,000,000	6	18.3 ±0.6
10,000,000	7	14.8 ±0.1
100,000,000	8	10.4 ±0.1
1,000,000,000	9	8.0 ±0.4
10,000,000,000	10	5.3 ±0.1

Figure S2. Phylogenetic tree of infectious AiV-1 strains detected by ICC-RT-qPCR

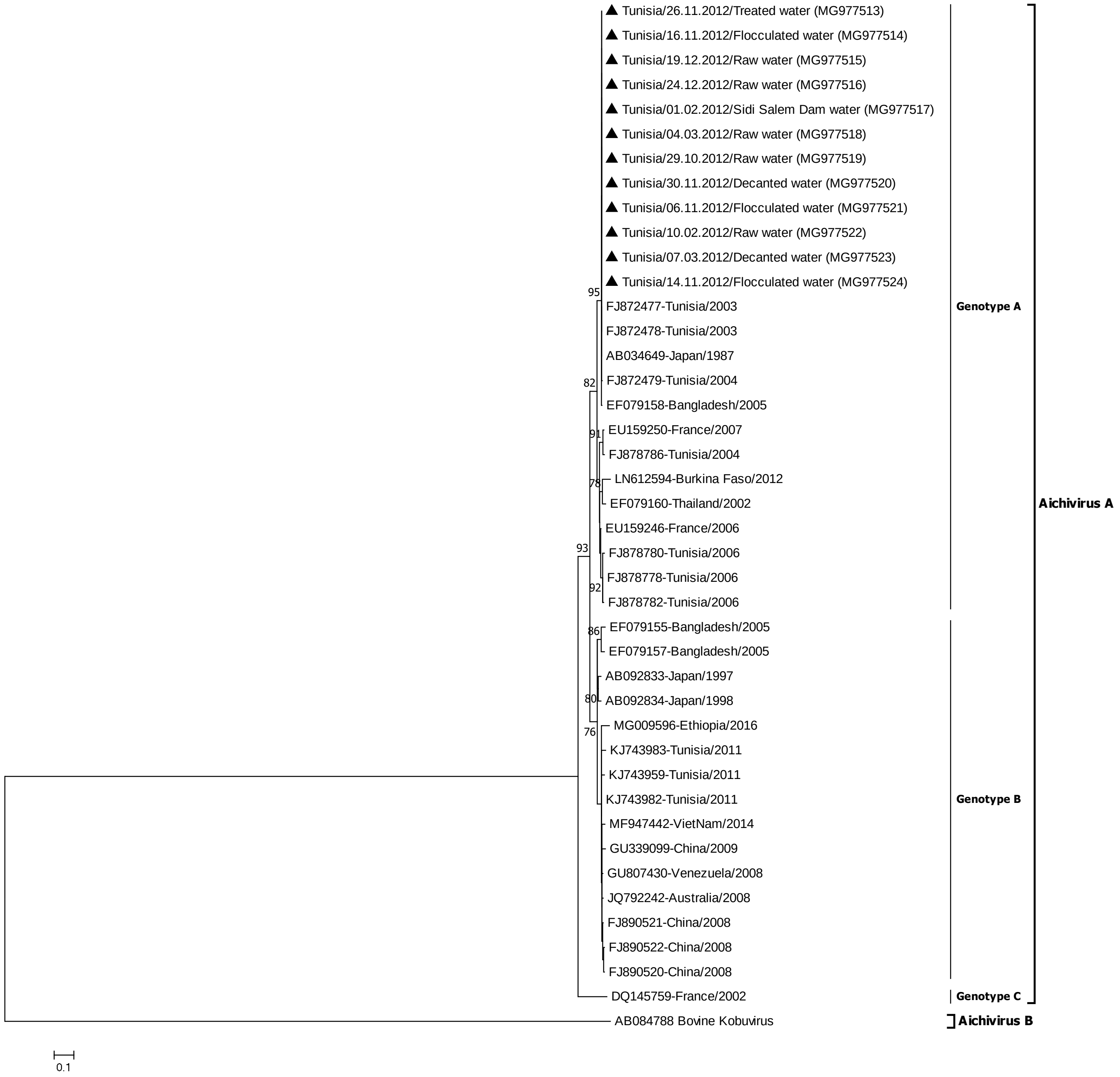

Table S1. Data summary of the 85 AiV-1-positive water samples

Sample water type	Sampling date	Ct	Log_{10} cp / L	*infective*	*Genbank number*
Raw Water (RW)	**2012-09-08**	**6.49**	**10.83**	*yes*	***MG977519***
	2012-02-16	10.24	9.65	*no*	
	**2012-03-12**	**10.29**	**9.64**	*yes*	***MG977518***
	2012-12-05	11.91	9.13	*no*	
	2012-02-27	12.10	9.07	*no*	
	2012-03-03	13.38	8.67	*no*	
	2012-02-09	13.56	8.61	*no*	
	2011-11-23	13.65	8.58	*no*	
	**2012-02-24**	**13.75**	**8.55**	*yes*	***MG977522***
	2012-02-28	13.78	8.54	*no*	
	2012-12-26	14.25	8.39	*no*	
	2012-02-03	14.60	8.28	*no*	
	2012-04-02	15.33	8.05	*no*	
	**2012-12-19**	**15.63**	**7.96**	*yes*	***MG977515***
	2012-12-19	17.82	7.27	*no*	
	**2012-12-24**	**23.15**	**5.59**	*yes*	***MG977516***
	2011-10-18	29.53	3.59	*no*	
	2012-09-19	30.63	3.24	*no*	
	2011-12-30	30.90	3.16	*no*	
	2012-03-05	30.99	3.13	*no*	
	2012-03-03	33.04	2.48	*no*	
	2012-03-25	33.39	2.37	*no*	
	2011-10-18	35.09	1.84	*no*	
	2011-10-18	35.14	1.82	*no*	
	2012-09-19	35.80	1.61	*no*	
	2012-02-20	36.22	1.48	*no*	
	2011-10-18	37.42	1.10	*no*	
	2012-11-14	37.44	1.10	*no*	
	2012-12-17	38.15	0.87	*no*	
	2012-12-19	39.43	0.47	*no*	
Decanted Water (DW)	2012-01-24	4.01	11.62	*no*	
	2011-12-07	8.00	10.36	*no*	
	2012-01-25	13.89	8.51	*no*	
	2012-03-28	14.06	8.45	*no*	
	2012-01-12	15.88	7.88	*no*	
	2013-06-17	16.31	7.74	*no*	
	**2011-02-01**	**22.01**	**5.95**	*yes*	***MG977523***
	**28.03.2012**	**30.43**	**3.30**	*yes*	
	2012-03-05	30.55	3.27	*no*	
	2011-02-01	30.76	3.20	*no*	
	2011-02-10	32.15	2.76	*no*	
	2011-02-01	32.40	2.68	*no*	
	2012-03-27	32.87	2.53	*no*	
	2011-02-01	33.16	2.44	*no*	
	**2011-11-25**	**33.37**	**2.38**	*yes*	
	2011-02-01	33.98	2.19	*no*	
	2011-11-02	36.73	1.32	*no*	

Sample water type	Sampling date	Ct	Log_{10} cp / L	*infective*	*Genbank number*
**Floculated Water (FW)**	2012-08-22	6.84	10.72	*no*	
	**2012-11-28**	**7.21**	**10.61**	*yes*	*MG977521*
	**2012-11-28**	**12.69**	**8.88**	*yes*	*MG977520*
	2012-08-13	13.83	8.52	*no*	
	2011-12-21	14.30	8.38	*no*	
	2012-02-10	14.30	8.38	*no*	
	2012-02-10	14.39	8.35	*no*	
	2012-12-17	14.60	8.28	*no*	
	2012-11-23	14.68	8.26	*no*	
	**2012-12-05**	**14.77**	**8.23**	*yes*	*MG977524*
	2011-12-21	27.56	4.20	*no*	
	**2012-11-28**	**28.42**	**3.93**	*yes*	*MG977514*
	2011-12-09	30.23	3.37	*no*	
	2012-10-03	30.36	3.33	*no*	
	2012-08-15	37.64	1.03	*no*	
**Treated Water (TW)**	2012-11-16	13.58	8.60	*no*	
	2012-11-08	17.23	7.45	*no*	
	2012-12-19	17.40	7.40	*no*	
	**2012-11-26**	**20.46**	**6.44**	*yes*	*MG977513*
	2011-02-07	27.74	4.15	*no*	
	2011-10-18	31.01	3.12	*no*	
	2011-10-18	33.83	2.23	*no*	
	2012-10-08	35.98	1.56	*no*	
	**2012-12-26**	**37.23**	**1.16**	*yes*	
**Treated Tap Water (TTW)**	2012-02-20	16.51	7.68	*no*	
**Sidi Salem Dam Water (SSD-W)**	2011-11-01	9.39	9.92	*no*	
	2011-12-13	11.22	9.34	*no*	
	2011-12-01	11.25	9.33	*no*	
	2012-02-14	20.55	6.41	*no*	
	2011-12-24	25.13	4.97	*no*	
	2012-11-24	25.33	4.91	*no*	
	2012-02-14	27.40	4.26	*no*	
	2011-12-08	27.47	4.23	*no*	
	2012-01-06	27.64	4.18	*no*	
	**2012-02-03**	**28.89**	**3.79**	*yes*	*MG977517*
	2011-12-24	31.12	3.09	*no*	
	2012-10-20	32.11	2.77	*no*	
	2012-11-20	35.59	1.68	*no*	
